# Distinctive origin of artemisinin-resistant *Plasmodium falciparum* on the China-Myanmar border

**DOI:** 10.1038/srep20100

**Published:** 2016-02-02

**Authors:** Run Ye, Dongwei Hu, Yilong Zhang, Yufu Huang, Xiaodong Sun, Jian Wang, Xuedi Chen, Hongning Zhou, Dongmei Zhang, Mathirut Mungthin, Weiqing Pan

**Affiliations:** 1 Department of Tropical Infectious Diseases, Second Military Medical University, Shanghai 200433, China; 2 Yunnan Institute of Parasitic Diseases, Puer 665000, China; 3 Department of Parasitology, Phramongkutklao College of Medicine, Bangkok 10400, Thailand

## Abstract

The artemisinin (ART), discovered in China, has been widely used against malaria in China over the last 30 years. Understanding the emergence and origin of ART resistance in China is therefore of great interest. We analyzed 111 culture-adapted isolates of *P. falciparum* from China-Myanmar (CM) border for their susceptibility to dihydroartemisinin using the ring stage survival assay (RSA_0−3h_) and genotyped their K13 genes. Of the isolates, 59 had a wild type of the K13 marker and a median ring survival rate of 0.26% (P_95_ = 1.005%). Among the remaining isolates harboring single mutations in the K13 marker, 26 survived at >P_95_(median survival rate = 2.95%). Further, we genotyped the K13 gene of 693 isolates collected from different regions in China and China-Myanmar/Thai-Cambodia/Thai-Myanmar (CM/TC/TM) borders, 308 (44.4%) had K13 mutations and marked differences in the patterns of K13 mutations were observed between the CM and the TC/TM borders. A network diagram showed that majority of the K13 mutant alleles from the CM border clustered together including those harboring the high resistant-associated R539T mutations. The resistant parasites carrying distinct halplotypes suggested the multiple indigenous origins of the resistant alleles, which highlight the importance of surveillance of resistance in all malaria endemic areas where ART has been introduced.

Artemisinin (ART) is a novel antimalarial drug discovered in China that is quick, effective, and has few adverse side-effects^1^,^2^. The World Health Organization (WHO) has recommended ART-based combination therapies (ACT) as first-line drugs for the treatment of *Plasmodium falciparum* malaria in all malaria endemic countries^3^. This has contributed to the recent significant decline in the global malaria burden^4^. Resistance to ART in *P. falciparum* is characterized by slow parasite clearance in patients receiving ART or an ACT^5^. ART resistance was first detected along the Thai-Cambodian border and has been detected across mainland Southeast Asia^5^,^6^,^7^,^8^,^9^,^10^. The continued spread of ART resistance will threaten malaria control programs globally. Therefore, the WHO has established a Global Plan for Artemisinin Resistance Containment (GPARC) to halt the spread of the resistance^11^. To this end, it is important to identify new areas where ART resistance is prevalent in order to implement containment intervention. ART and its derivate drugs were discovered in China and have been widely used there for the past 30 years^1^,^2^. Nevertheless, little is known about the patterns of emergence and distribution of ART resistance in China, particularly on the southern border. Understanding whether ART resistance has spread to China, or independently emerged in China, is thus, of great interest.

In attempts to contain the ART resistance, the prompt and effective detection and monitoring of resistant parasites is a huge challenge. However, significant progress has recently been made in this respect. First, a novel *in vitro* method has been established that can distinguish culture-adapted parasites from patients with slow-clearing or fast-clearing infections (based on a ring stage survival assay of 0 to 3 h ring stage parasites; ‘RSA_0–3h_’). The survival rates from RSA_0–3h_ are strongly correlated with *in vivo* parasite clearance rates^12^. Second, the propeller domain of the K13 gene of *P. falciparum* has been identified as a molecular marker for the detection and monitoring of ART-resistant parasites. Mutations in this region of the K13 gene are associated with slow parasite clearance rates *in vivo* and high parasite survival rates *in vitro*^13^. A recent study showed that mutations in the propeller domain of the K13 gene could identify a parasite clearance half-life of >5 hours with 91.8% sensitivity and 88.4% specificity^10^. The RSA_0–3h_ method and the K13 marker offer several advantages over *in vivo* measurement. of parasite clearance rates as they minimize the influence of confounding factors, including variation in host immunity, drug dosage, and drug absorption and metabolism^12^. Thus, both assays should provide the effective and prompt monitoring of *P. falciparum* isolates for their susceptibility to ART. In addition, recent studies identified several genetic markers that can distinguish the K13 mutant alleles based on their geographic locations^14^,^15^,^16^. Therefore, in this study we use these two assays to investigate if the ART-resistant malaria parasites emerged in China and its relevant border districts, and use additional genetic markers to trace the origins and evolution of the K13 mutant alleles.

## Results

### Spatial variation in K13 genotypes

**Figure 1:**
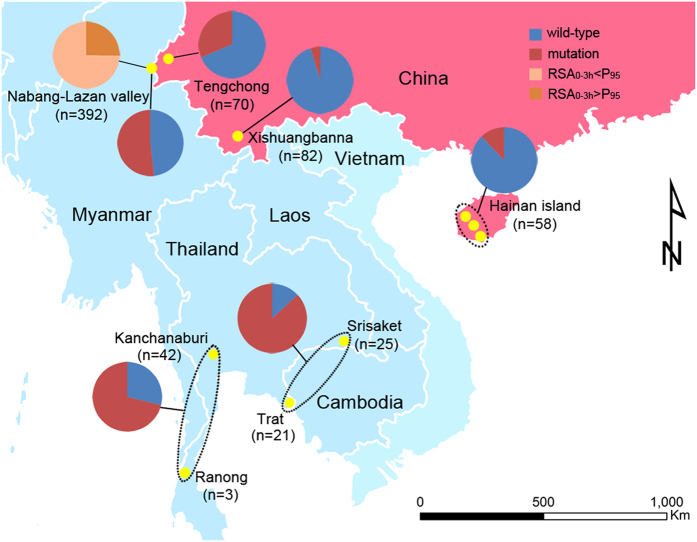
Map showing location of study sites and proportions of isolates with high survival rates and mutations of the K13 gene. The proportions of isolates with parasite survival rates above P_95_ are indicated in orange and below P_95_ in pink in the Nabang-Lazan valley. The proportions of wild type isolates are shown in blue and with mutations in the propeller domain of the K13 gene in red. The map was created in ArcGIS 9.3 software (ESRI Inc., Redlands, CA, USA) (http://www.esri.com/).

We genotyped the K13 gene of 602 *P. falciparum* isolates collected from different regions in southern China and the CM border and 91 isolates from the TC/TM (Thai-Cambodia/Thai-Myanmar) borders (Fig. 1). Of the 602 isolates from southern China and the CM border, 366 isolates (60.8%) with a wild type K13 allele and 236 isolates (39.2%) with a single mutation in the propeller domain of the gene (Table 1). We identified 31 non-synonymous SNPs, 15 of which were new mutations. The frequencies of the SNPs varied spatially. All but seven of the 58 isolates collected from Hainan Island had a wild type K13 allele. Those seven isolates possessed a single mutation in the propeller domain, including the A481V, P553L and H719N mutations associated with slow parasite clearance^10^ or higher ring stage survival rates. Moreover, only four of the 82 isolates collected from XSBN (Xishuangbanna) in Yunnan Province in 2003 and 2004 had a single mutation in the domain, revealing very low frequency of the K13 mutant alleles. In contrast, over half of the 392 isolates from the CM border possessed single mutations in the domain. Interestingly, the frequency of these mutant alleles tended to increase over time (Supplementary Figure S1). Further, we found marked differences in the patterns of K13 mutations between the CM and the TC/TM borders (Table 1). The most common mutation from the CM border (F446I with a frequency of 32.7%) was absent in the TC/TM borders. In contrast, the C580Y mutant was common in the TC/TM borders (frequency = 54%), but only present in two of the 602 isolates from the CM border and southern China. Importantly, we found 16 isolates with the R539T mutation that was strongly associated with the higher ring stage survival rates and previously shown to be associated with delayed parasite clearance rates *in vivo*^13^.

### ART susceptibility of field isolates by RSA_0−3h_

**Figure 2:**
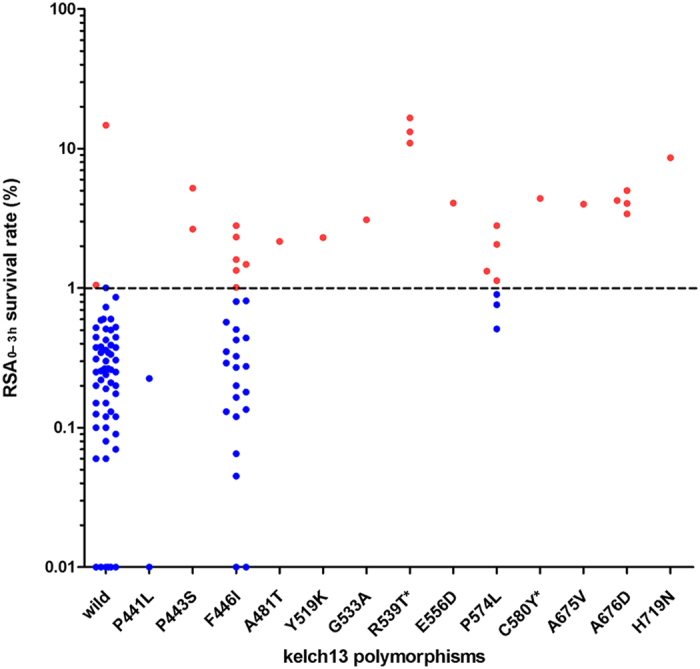
Correlation of parasite survival rates in the RSA_0−3h_ and K13 polymorphisms in 111 isolates. Red circles: survival rates >P_95_; blue circles: survival rates <P_95_. One circle represents one isolate. The locations of the K13 mutations are indicated as amino acid positions. Mutations previously reported to be associated with ART resistance are indicated by an asterisk.

Next we measured the sensitivity of the field isolates to ART by the RSA_0−3h._ The result showed that the ring stage survival rates of the 111 isolates collected from the CM border ranged from 0.01% to 16.6%, revealing marked differences in the survivorship of field collected isolates exposed to ART (Fig. 2). Of 111 isolates collected from the CM border, 59 isolates harbored the wild type of K13 gene and had median ring stage survival rate of 0.26% (IQR (interquartile ranges), 0.12–0.43%, P_95_ = 1.005%). We defined an RSA_0−3h_ rate greater than the P_95_ value as ‘higher ring stage survival rate’. Among the remaining 52 isolates that harbored a single mutation in the K13 propeller domain, half (N = 26) had a higher ring stage survival rate (median = 2.95%, IQR = 1.95% to 4.55%), four of which survived at >10% (Fig. 2). The remaining isolates (N = 26) had ring stage survival rates lower than the P_95_ value. The three isolates with the R539T mutation, which was strongly associated with the delayed parasite clearance rates *in vivo*, also had the highest levels of ring stage survival rates (16.6%, 13.16% and 10.95%). Isolates with K13 mutant alleles in the propeller domain exhibited higher ring stage survival rates, except for those that contained the F446I mutant allele (N = 27) or the P441L mutant allele (N = 2). The mean RSA_0−3h_ rate of the 27 isolates with the F446I mutation was 0.62%. Of these, 21 isolates had survival rates <P_95_ value. There was no significant difference in the RSA_0−3h_ rates between the F446I mutant alleles and the wild type alleles (Mann-Whitney U test, P = 0.16), suggesting that F446I mutant allele is not associated with higher ring stage survival rates. Excluding the F446I and P441L mutant alleles, the sensitivity and specificity of the remaining mutations to identify parasites with higher ring stage survival rates were 90.9% and 95%, respectively.

### Origin and evolution of the K13 mutants

**Figure 3:**
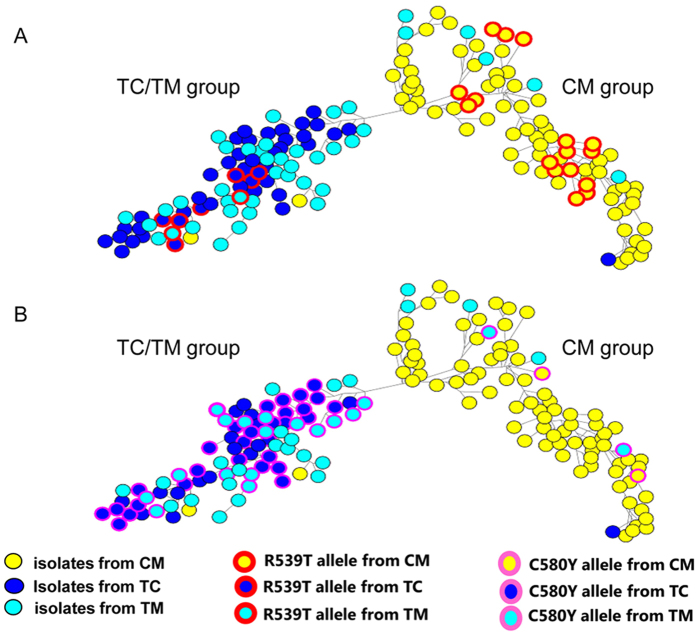
Network diagram revealing three geographically distinct origins for the R539T and C580Y mutant alleles. Based on data from 11 neutral microsatellites and three SNPs (*arps10*, *fd* and *pph*), all 169 isolates were initially classified into two major independent networks, e.g. China-Myanmar(CM) group and Thailand-Cambodia/Thailand-Myanmar(TC/TM) group. Isolates from the CM border, the TC border and the TM border are shown in yellow, dark blue and sky-blue respectively. Dots with red and pink circles represent the isolates with K13 mutant alleles R539T and C580Y respectively. (**A**) origins of the R539T mutant allele, (**B**) origins of the C580Y mutant allele.

We identified 18 isolates with the R539T and C580Y mutations from the CM border. To determine whether these highly ART resistance-associated mutants evolved locally or spread from other areas, such as the epicenter of artemisinin resistance, we genotyped 169 isolates (including 82 from the CM border and Tengchong, 46 isolates from the TC border and 41 isolates from the TM border) at 11 neutral microsatellite loci (Supplementary Table S1) and three SNPs (*fd* (ferredoxin), *arps10* (apicoplast ribosomal protein S10) and *pph* (Protein phosphatase) in Supplementary Table S2) recently identified to be associated with the resistant founder populations^15^. A median-joining network diagram tree generated from these haplotypes showed that these parasites were structured according to their geographic origins: one group consisting of samples from the CM border and a second group comprising isolates from the TC/TM borders (Fig. 3). Only six isolates from the TM border and one isolate from the TC border clustered with the CM group, while two isolates from the CM border clustered with the TC/TM group (Fig. 3). Importantly, all the parasites harboring the R539T mutation that were collected from the CM border clustered in the CM group, while those mutants collected from the TC/TM borders clustered in the TC/TM group (Fig. 3A). This suggests that the R539T mutant alleles had multiple distinct origins. In addition, the majority of the isolates with the other K13 mutations, including those with higher ring stage survival rates identified in this study, clustered together in the CM group. This suggests that the majority of isolates with moderate resistant-associated mutations evolved locally.

**Figure 4:**
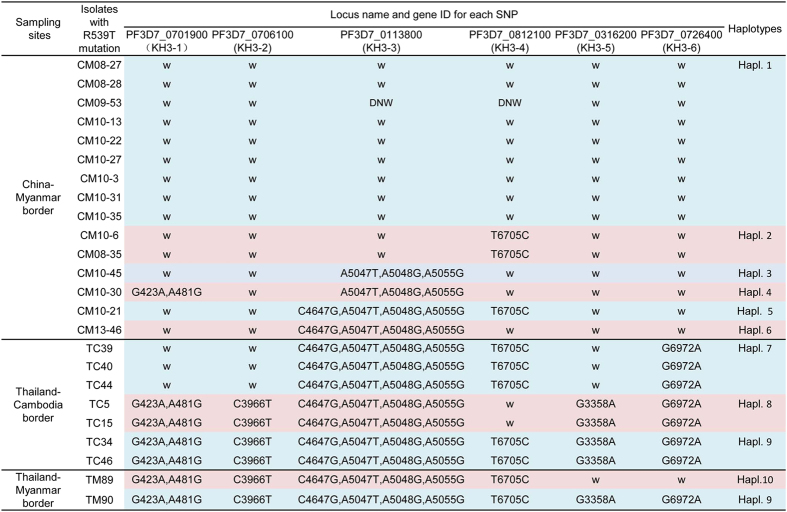
Haplotypes of the isolates with the R539T mutation from the China-Myanmar border, Thailand-Cambodia border and Thailand-Myanmar border. The ten SNP markers are located in the six genes (KH3 1–6) that highly differentiated in KH3 subpopulation of Cambodia, where artemisinin-resistant isolates with R539T mutations are found. Haplotypes are defined based on the combination of the SNPs. w: wild type, DNW: no successful amplification.

To further verify the origin and evolution of the R539T mutant alleles, we used a set of SNP markers that were recently reported to differentiate the Cambodian ART-resistant founder subpopulations (i.e. KH2, KH3 and KH4) from the other populations^14^. As the majority of the R539T mutant isolates were located in the KH3 subpopulation, we selected ten SNP markers from this subpopulation to trace the origin of all the mutant alleles identified from this study (Supplementary Table S2). A total of ten halplotypes were identified for all the isolates. Overall, this combination of SNP markers clearly differentiated R539T mutants from the CM border and mutants from the TC/TM borders (Fig. 4). Haplotypes 1 to 6 were found only in the isolates from the CM border, whereas haplotypes 7 to 10 were unique to isolates from the TC/TM borders. Interestingly, of the 15 isolates with R539T allele from the CM border, nine had the wild type allele for all the ten SNP markers, except two SNPs in the CM09-53 isolate that did not amplify successfully after several attempts (Fig. 4). Moreover, the G6972A SNP in the PF3D7_0726400 gene was able to separate R539T mutant isolates into their geographic locations (CM versus the TC borders).

The neighbor joining tree analysis showed that the two isolates with C580Y mutations (CM09-15 from the Nabang-Lazan valley on the CM border and TEC43 from Tengchong in Yunnan province) clustered with parasites from the CM border. Two other isolates (TM1 and TM26) with the C580Y mutation collected from the TM border (Kanchanaburi province in Western Thailand), however, also clustered with the CM group of parasites (Fig. 3B). This suggested that the two isolates (CM09-15 and TEC43) with C580Y mutant allele from the CM border and Tengchong may have spread from the TM border. To address this possibility, we used two microsatellite loci, which flank the K13 gene (8.6 kb and 31.5 kb upstream and downstream of the K13 gene) and were recently reported to separate C580Y alleles geographically^16^. A total of five haplotypes were identified from all the parasites with the C580Y mutations (Supplementary Table S3). Consistent with previous findings^16^, all the Thai-Cambodia C580Y alleles had a common haplotype (Hapl.1 in Supplementary Table S3) with a 288 bp allele size at locus 8.6kb and 194 bp at locus 31.5kb. However, the Thai-Myanmar C580Y alleles carried several different haplotypes (Hapl.1 to 5 in Supplementary Table S3). Importantly, the two C580Y mutants from the CM border and Tengchong carried haplotypes 2 and 5 respectively, which were found only in the parasites from the TM border but not in parasites from the CM border (Supplementary Table S3). This suggests that the two CM C580Y mutants (CM09-15 and TEC43) can be traced to an ancestor originating in the TM border.

## Discussion

The antimalarial drug ART was derived from the Chinese medical herb *Artemisia annua*, which had for centuries been used to treat fever and malaria in China^1^,^2^. A series of clinical trials of the drug began in the mid-1970s while ART-based therapies were introduced in the 1980s when the parasite had developed resistance to chloroquine and sulfadoxine/pyrimethamine in southern China^17^,^18^. Before 2005, the ART was widely used in Yunnan province and on China-Myanmar border mostly as monotherapy. For example, artemtherin was introduced in the 1980s while artesunatum and dihydroartemisinin were used as monotherapy since 1990s^19^. Several clinical trials began in 2003 for testing dihydroartemisinin/piperaquine (DHP) combination in Yunnan province while the DHP was widely used in this area for the treatment of *falciparum* malaria since 2005, compared to the artesunate/mefloquine combination used in other areas in Southeast Asia. For a number of reasons, such as the long-term, mostly as monotherapy, perhaps unregulated and widespread use of ART-based therapies, much attention has been given to the emergence of ART-resistant parasites in China. In this study, we report the emergence of ART resistance in *P. falciparum* along the China-Myanmar border and southern China. In particular, 24% of the 111 isolates we examined had a resistant phenotype and exhibited higher ring stage survival rates. The median survival rates of these resistant isolates was 2.95%, though we observed four parasite isolates with survival rates >10% when exposed to ART. In addition, we identified 18 isolates with mutations associated with high ART-resistant phenotype from the China-Myanmar border (16 with the R539T mutation and two with the C580Y mutation)^13^. Importantly, these parasites with the highly ART resistance-associated mutations such as R539T allele carried distinct halplotypes from those of parasites found along the Thai-Cambodia borders, suggesting that the K13 mutant alleles evolved locally. Thus, our results indicate that ART-resistant parasites have spread throughout the Greater Mekong Subregion (GMS) of Southeast Asia. This situation is similar to resistance to chloroquine and sulfadoxine/pyrimethamine, which emerged in the Asia-Pacific region in the 1970s and spread to Africa, significantly worsening malaria care regionally^20^. Therefore, the multiple origins and spread of the ART resistance throughout the whole GMS should be taken seriously. The findings from this study could be used to inform national malaria control programs and the WHO/GPARC in terms of the expansion or intensification of containment measures.

The RSA_0−3h_ is a recently developed *in vitro* assay capable of distinguishing *in vitro* adapted isolates with fast parasite clearance from those with slow clearance. ART resistance correlates strongly with the ring stage survival rate *in vitro*^12^. Previous studies have shown that RSA_0−3h_-determined resistance phenotypes are strongly associated with mutations in the propeller domain of the K13 gene^13^. The data from this study similarly revealed a strong association between higher ring stage survival rates (RSA_0−3h_-determined) and mutations in the K13 gene propeller domain. For example, the R539T and C580Y mutant alleles associated with delayed parasite clearance rates *in vivo* exhibited increased RSA_0−3h_ rates with mean survival rate greater than 10%. In contrast, the isolates containing the wild type K13 gene had a median RSA_0−3h_ rate of only 0.26%. Hence, the data from this study further support the use of both RSA_0−3h_ and the K13 marker for the effective and rapid detection of ART resistance in *P. falciparum*.

We found that the F446I mutant was not statistically associated with higher ring stage survival rates compared to the RSA_0−3h_ rate of wild type alleles. The median survival rate of all the 27 isolates with the F446I mutation was only 0.33% (IQR 0.14-0.81), although five of the isolates had survival rates greater than the P_95_ value (Fig. 2). This result was consistent with previous studies showing that the majority of patients with this mutant allele had a parasite clearance half-life below five hours, suggesting this mutation should not be associated with ART resistance^10^,^15^. However, a recent study using a large sample size reported that the F446I mutation was associated with prolonged parasite clearance half-life^21^. In this study, the parasitemia was measured every 12 hours rather than typical measurement of every 6 hours, which may have inflated the parasite clearance half-life^21^. The F446I mutation is the most prevalent allele in China-Myanmar and in the north of Myanmar including sites very close to the Indian border^21^,^22^,^23^. Therefore, further investigation is needed to clarify whether the F446I mutation is an ART-resistant marker.

Our survey of 602 isolates collected from different malaria endemic areas in China and along the Chinese border indicated that 39.2% of isolates possessed a single mutation after position 440 in the K13 gene, with evident regional differences in the prevalence of this mutation. More than half the isolates from the China-Myanmar border harbored the K13 mutations, but only 4.9% of isolates from XSBN and 12% from Hainan harbored the mutation. The F446I mutation was the most common mutation along the China-Myanmar border (32.7%) and in Tengchong (14.3%), but the frequency of this mutation was extremely low in the other areas of Southeast Asia (0.01%)^10^. Moreover, we identified 15 new non-synonymous mutations of the K13 gene which were not reported in the other areas of Southeast Asia. These data suggest that the parasites from the China-Myanmar border and Yunnan province exhibited a different pattern of K13 polymorphism from those in other areas of Southeast Asia.

Hainan Island is a major endemic region of *P. falciparum* malaria in China. ART-based therapies have been used on the island for clinical trials and for treatment of chloroquine-resistant malaria since the 1980s. Despite the long history of ART on the island, we found that the prevalence of the K13 mutation was generally low (12%). Nevertheless, we found resistance-associated mutations of the K13 propeller domain in seven isolates collected as early as 2005. This is the first report of these K13 mutant alleles from Hainan Island. Importantly, three of the mutations (A481V, P553L and H719N) were previously reported to be strongly associated with ART resistance^10^. These findings suggest that ART-resistant parasites either emerged, or spread to, this island as early as 2005. Interestingly, despite the presence of ART-resistant parasites, the malaria burden on Hainan Island has declined significantly over the past ten years^24^.

Recently, several patients were identified who exhibited slow parasite clearance rates (half-life >5 hours) but did not have the phenotype-associated K13 mutation (wild type)^10^. In this study, we similarly identified one isolate (CM09-16) that harbored a wild type allele of the K13 gene yet exhibited a high survival rate (14.7%). The lack of the association of the resistance phenotypes with K13 polymorphism in some field isolates suggests that additional molecule(s) are involved in the development of ART resistance in *P. falciparum*. Further research is needed to identify these genetic determinants of the resistant phenotype.

The R539T mutant allele was strongly associated with ART resistance characterized by both delayed parasite clearance rates *in vivo* and higher ring stage survival rates *in vitro*^10^,^13^. This allele was originally identified from a central hotspot of high-frequency resistance in western Cambodia^13^. Recent investigations have described multiple origins for several K13 mutant alleles, including C580Y but excluding the R539T allele^23^. Moreover, this allele was absent in 55 studied sites in Myanmar^23^, and there is no evidence that the resistant allele has spread from Cambodia into Myanmar^25^. However, we identified 16 isolates with the R539T in the Nabang-Lazan valley on China-Myanmar border. We found that isolates with the R539T mutation clustered together based on their geographic locations using the neutral microsatellite loci and the SNPs that were associated with the resistant founder populations, suggesting that these 16 isolates evolved independently to those from the TC/TM borders.

In addition to the mutations in the K13 locus, SNPs such as fd-D193Y (Asp193Tyr substitution in ferredoxin), arps10-V127M (Val127Met substitution in apicoplast ribosomal protein S10) and pph-V1157L (Val1157Leu substitution in protein phosphatase) are associated with ART resistance and have close to 100% frequency in isolates containing the R539T mutation^15^. Consistent with this finding, we found that all the R539T mutant parasites from TC/TM border had both fd-D193Y and arps10-V127M mutations. Surprisingly, we found that both fd-D193Y and arps10-V127M mutant alleles were absent from the majority of R539T mutants from the CM border (three isolates had the fd-D193Y mutation) (Supplementary Table S4). Furthermore, analyses with 10 additional SNP markers of the KH3 subpopulation^14^, we could differentiate the R539T mutant alleles from the CM and the TC/TM borders. Also, the R539T mutant parasites had geographically distinct haplotypes. Taken together, all these results suggest that the R539T mutant alleles from the CM border evolved locally and thus, revealing the possibility of the independent evolution of the R539T mutant allele. This possibility suggests that efforts to prevent the spread of resistance should focus on preventing the spread of resistance from all emergent areas and highlights the importance of surveillance of resistance in all malaria endemic areas.

## Methods

### Study sites and sample

All the parasites in this study were collected from symptomatic malaria patients that were positive for *P. falciparum* by Microscope. The patients involved in this study were treated with dihydroartemisinin and piperaquine phosphate combination after sampling. The majority (N = 392) of the *P. falciparum* isolates were collected from a valley populated by approximately 50,000 people located on the China-Myanmar (CM) border (longitude: 97°33′47.81″; latitude: 24°45′21.65″) from 2007 to 2013. The border follows a stream through the valley, named ‘Nabang’ on the China side and ‘Lazan’ on the Myanmar side (hereafter named the ‘Nabang-Lazan valley’). Of the isolates, 95 had the parasite clearance time data (Supplementary Table S5). In addition, we obtained parasite isolates from Tengchong (TEC; N = 70, collected 2006–2007) and Xishuangbanna (XSBN; N = 82, collected 2003–2004) in Yunnan Province and from Hainan Island (HN; N = 58, collected 2004–2008) in Southern China (Fig. 1). To identify the origins of the artemisinin-resistant parasites, we collected a further 45 isolates of *P. falciparum* from Kanchanaburi and Ranong along the Thai-Myanmar (TM) border in 2012–2014, and another 46 isolates of *P. falciparum* from the townships of Borai/Trat, Klongyai/Trat and Srisaket along the Thai-Cambodia (TC) border in 2009–2013 (Fig. 1). (see supplementary methods). Informed consent was obtained from the patients and all experiments were performed in accordance with relevant guidelines approved by the Internal Review Board of Second Military Medical University.

### Parasite culture and ring stage survival assay (RSA_0−3h_)

The isolates collected from malaria patients were first established for continuous cultivation *in vitro*^26^. The culture-adapted parasites were synchronized two to three times to obtain tightly synchronous parasites. The RSA_0−3h_ was carried out as previously described^12^. (see supplementary methods)

### DNA isolation and Genotyping of the K13 gene and SNP markers

DNA was extracted from venous blood samples or finger prick blood on filter paper using QIAmp Mini kits (Qiagen, Germany). Either the entire or propeller domain of *P. falciparum* K13 gene (from nucleotide position 1279 to 2181) were obtained by means of a nested PCR according to published methods^10^,^13^. PCR products were sequenced and these sequences were aligned to the gene of the *P. falciparum* 3D7 isolate to identify single nucleotide polymorphisms (SNPs) with MEGA5 software version 5.10.

The SNP markers in *fd*, *arps10* and *pph* were strongly associated with parasite clearance half-life (PC t1/2) as described^15^. The isolates with R539T mutation were mainly found in KH3 sub-populations in western Cambodia and the SNPs that highly differentiated in this cluster were also selected^14^. The information of Gene ID, SNPs and primers were shown in Supplementary Table S2. The cycling parameters were: 94 °C for 2 min; 30 cycles of 98 °C for 30 s, 58 °C for 30 s, and 68 °C for 30 s; and 68 °C for 5 min. PCR products were sequenced and aligned with the standard gene sequences of 3D7 clone to indentify the SNPs.

### Microsatellite analysis

Eleven neutral microsatellite markers, including ARA2, TA1, TAA42, TAA60, TAA81, TAA87, TAA109, pfG377, pfPK2, Polyα, and B5M2 were selected for analysis of their diversities^27^,^28^. These microsatellite markers are located in different chromosomes. The length of the microsatellites varied from 70 bp to 224 bp with the reiterating sequence as long as three nucleotides. The description of microsatellite loci, primers and PCR parameters are given in Supplementary Table S1, except B5M2. This Locus had been identified recently and added to this study. The primers and amplification conditions used to genotype the microsatellite loci (8.6kb, 31.5kb) flanking the K13 gene are from the previous study^16^. Variation in the length of PCR products was measured using QIAxcel (QIAGEN, Switzerland). Fragment size polymorphism was analyzed using the Bio Calculator software.

### Statistical analyses

Quantitative data were expressed as medians with interquartile ranges (IQR). We assumed that values below the 95th percentile (P_95_) of the survival rates of the parasites with wild type of the K13 gene were ‘normal’. The Mann-Whitney U test was used to compare the survival rate of the control and the treatment groups with differences considered statistically significant at P < 0.05. Data was analyzed with Microsoft Excel, GraphPad Prism 5.0 and IBM SPSS 19.0. To identify the origin and characterize the evolution of the R539T and C580Y mutant alleles, we constructed a median-joining tree based on 11 neutral microsatellite loci combined with three SNPs using the program NETWORK (version 4.613, http://www.fluxus-engineering.com/sharenet.htm). In addition, to determine the exact number of origins, genotypic data based on SNPs was converted into PCA (Principal Component Analysis, PCA) data using program SIMCA-P (version 11.5).

## Additional Information

**How to cite this article**: Ye, R. *et al*. Distinctive origin of artemisinin-resistant *Plasmodium falciparum* on the China-Myanmar border. *Sci. Rep*. **6**, 20100; doi: 10.1038/srep20100 (2016).

## Supplementary Material

Supplementary Information

## Figures and Tables

**Table 1:** Polymorphisms observed in the K13 gene in *P. falciparum* isolates collected from Hainan, Yunnan (Tengchong and Xishuangbanna), China-Myanmar, Thailand-Myanmar and Thailand-Cambodia.

Mutations name	Hainan	Tengchong	Xishuangbanna	China- Myanmar	Thailand-Myanmar	Thailand-Cambodia
Wildtype	51	48	78	189	13	6
P441L	0	0	0	4	0	0
P443S	0	0	0	2	0	0
F446I	0	10	0	128	0	0
Q468K*	0	0	0	1	0	0
C469Y	0	0	0	3	0	0
A481T*	0	0	0	1	0	0
A481V	2	0	0	1	0	0
F483S	0	0	0	2	0	0
F491L*	0	0	0	2	0	0
L492S	0	0	0	1	0	0
Y493H	0	0	0	0	0	2
F495S*	0	0	0	1	0	0
Y519K*	0	0	0	1	0	0
G533A	0	0	0	2	0	0
G538V	0	0	0	0	3	2
R539T	0	0	0	16	2	7
P553L	1	3	0	2	0	0
E556D	0	0	0	4	0	0
R561H	0	0	0	0	1	0
P574L	0	3	0	14	4	0
S577P*	0	0	1	0	0	0
C580Y	0	1	0	1	20	29
T593N*	0	0	0	1	0	0
E605K*	0	1	0	0	0	0
P615T*	0	0	0	1	0	0
P615Q*	0	0	0	1	0	0
G625V*	0	0	1	0	0	0
P667Q*	0	0	1	0	0	0
F673L*	2	0	1	1	0	0
A675V	0	0	0	2	2	0
A676D	0	3	0	6	0	0
S711T*	0	0	0	1	0	0
W718C*	1	0	0	0	0	0
H719N	1	1	0	4	0	0
Total	n = 58(12%)	n = 70(31.4%)	n = 82(4.9%)	n = 392(51.8%)	n = 45(71.1%)	n = 46(87%)

The proportion of mutant alleles in each site was calculated in parentheses.

*newly reported.
