# Supplementary material for: Distinctive origin of artemisinin-resistant Plasmodium falciparum on the China-Myanmar border
Source: Sci Rep. 2016 Feb 2;6:20100. doi: 10.1038/srep20100 (PMC4735722; doi:10.1038/srep20100)
Supplement: Supplementary Information [file srep20100-s1.doc]

**Supplementary Information**

Distinctive origin of artemisinin-resistant *Plasmodium falciparum* on the China-Myanmar border

Run Ye^1#^, Dongwei Hu^1#^, Yilong Zhang^1#^, Yufu Huang^1^, Xiaodong Sun^2^, Jian Wang^2^, Xuedi Chen^1^, Hongning Zhou^2^, Dongmei Zhang^1*^, Mungthin Mathirut^3^, Weiqing Pan^1*^.

^1^ Department of Tropical Infectious Diseases, Second Military Medical University, Shanghai, China;

^2^ Yunnan Institute of Parasitic Diseases, Puer, China;

^3^ Department of Parasitology, Phramongkutklao College of Medicine, Bangkok, Thailand.

* Correspondence: Dr. Weiqing Pan or Dr Dongmei Zhang, Department of Tropical Infectious Diseases, Second Military Medical University, Shanghai, China. (wqpan0912@163.com or dmzhangcn@163.com). Tel: 86-21-81871010; Fax: 86-21-65331272.

**Supplementary Table S1. Information of microsatellite markers, primer sequences and amplification conditions.**

| Name | Chr. | Repeats | Bp length | Sequence |
| --- | --- | --- | --- | --- |
| TA1-3(F) | 6 | (AAT)_8-15_ | 168 | CATCATGCCTAATGAGCA |
| TA1-R |  |  |  | TTTTATCTTCATCCCCAC |
| TA1-F |  |  |  | CCGTCATAAGTGCAGAGC |
| Polyα-R | 4 | (AAT)_11-20_ | 224 | ATCAGATAATTGTTGGTA |
| Polyα-F |  |  |  | AAAATATAGACGAACAGA |
| Polyα-3(IR) |  |  |  | GAAATTATAACTCTACCA |
| PfPK2-3(R) | 12 | (TTA)_8-13_ | 168 | CCTCAGACTGAAATGCAT |
| PfPK2-F |  |  |  | CTTTCATCGATACTACGA |
| PfPK2-R |  |  |  | AAAGAAGGAACAAGCAGA |
| TAA109-3(F) | 6 | (CTA)_6-12_ | 161 | TAGGGAACATCATAAGGAT |
| TAA109-R |  |  |  | CCTATACCAAACATGCTAAA |
| TAA109-F |  |  |  | GGTTAAATCAGGACAACAT |
| TAA81-3(F) | 5 | (AAT)_7-15_ | 124 | GAAGAAATAAGGGAAGGT |
| TAA81-R |  |  |  | TTTCACACAACACAGGATT |
| TAA81-F |  |  |  | TGGACAAATGGGAAAGGATA |
| TAA42-3(F) | 5 | (AAT)_7-8_ | 183 | ACAAAAGGGTGGTGATTCT |
| TAA42-R |  |  |  | GTATTATTACTACTACTAAAG |
| TAA42-F |  |  |  | TAGAAACAGGAATGATACG |
| ARA2-3(F) | 11 | (AAT)_9-12_ | 70 | GTACATATGAATCACCAA |
| ARA2-R |  |  |  | GCTTTGAGTATTATTAATA |
| ARA2-F |  |  |  | GAATAAACAAAGTATTGCT |
| TAA60-F | 13 | (AAT)_8-13_ | 202 | CTCAAAGAAAAATAATTCA |
| TAA60-R |  |  |  | AAAAAGGAGGATAAATACAT |
| TAA60-3(IF) |  |  |  | TAGTAACGATGTTGACAA |
| Pfg377-3(R) | 12 | (AAT)_7-9_ | 98 | TTATGTTGGTACCGTGTA |
| Pfg377-F |  |  |  | GATCTCAACGGAAATTAT |
| Pfg377-R |  |  |  | TTATCCCTACGATTAACA |
| TAA87-3(F) | 6 | (AAC)_7-11_/(AAT)_9-12_ | 112 | ATGGGTTAAATGAGGTACA |
| TAA87-R |  |  |  | ACATGTTCATATTACTCAC |
| TAA87-F |  |  |  | AATGGCAACACCATTCAAC |
| B5M2-F | 7 | / | 165 | TTATATACCCATGAAGGTAAT |
| B5M2-R |  |  |  | TCGGTGAATGATCATTCTAAT |
| B5M2-NF |  |  |  | ATCAATATTTTTCTCATTCTC |

All primers are listed 5’-3’. For each locus the first and second primers are used in the first round reaction, the second and third primers are used in the second round reaction.

**Supplementary Table S2. SNP markers and primers.**

| **Gene ID** | **SNPs** | **Primers(5’-3’)** | **length (bp)** | |
| --- | --- | --- | --- | --- |
| PF3D7_0701900 | G423A,A481G | KH3-1-F: CTATTTCTTCGTCGTCGTCTAC  KH3-1-R: TTGTACGTAATGATAACATGAAAC | 700 |  |
| PF3D7_0706100 | C3966T | KH3-2-F: CATGTTTCTATTCTCATTCGT  KH3-2-R: CTGAATTTTAATGAAGGGTT | 696 |  |
| PF3D7_0113800 | C4647G,A5047T,A5048G,A5055G | KH3-3-F: TTTTGGGCTTATTGTTTGG  KH3-3-R: TCATAAACGTAATCGTCCATA | 610 |  |
| PF3D7_0812100 | T6705C | KH3-4-F: CAAATGATTCGGGTGATA  KH3-4-R: GCTGTAAGTGTTAAATATGCAC | 784 |  |
| PF3D7_0316200 | G3358A | KH3-5-F: GTATTAATAGCGAACTAGCCA  KH3-5-R: AAAATTCAATACACTCACCAT | 498 |  |
| PF3D7_0726400 | G6972A | KH3-6-F: ATCTGAAGCATTATTAACCCT  KH3-6-R: CGTCGACATTTGTGCACAT | 373 |  |
| PF3D7_1460900.1 | G631A (arps10-V127M) | arps10-F: CCAAAAGACAATAAGAAAGAGG  arps10-R: AAGTAGTATTCAAAACCCACA | 414 |  |
| PF3D7_1318100 | C1001A (fd-D193Y) | fd-F: AATGAGATAAATGTGTTGGTC  fd-R: TTAGATGCTAGTGAAAGACAGA | 648 |  |
| PF3D7_1012700 | G3469C(pph-V1157L) | pph-F: AAAGTCAAGAGTCCTCTCA  pph-R: ATTATTGACTTCGTTCACATG | 533 |  |

**Supplementary Table S3. K13 flanking microsatellites of parasites from** **China-Myanmar border, Thailand-Myanmar border and Thailand-Cambodia border**

| sampling sites | sample | mutation | 8.6kb | 31.5kb | haplotype |
| --- | --- | --- | --- | --- | --- |
| Thai-Cambodia | TC1 | C580Y | 288 | 194 | Hapl. 1 |
|  | TC3 | C580Y | 288 | 194 |  |
|  | TC4 | C580Y | 288 | 194 |  |
|  | TC7 | C580Y | 288 | 194 |  |
|  | TC9 | C580Y | 288 | 194 |  |
|  | TC10 | C580Y | 288 | 194 |  |
|  | TC11 | C580Y | 288 | 194 |  |
|  | TC13 | C580Y | 288 | 194 |  |
|  | TC16 | C580Y | 288 | 194 |  |
|  | TC17 | C580Y | 288 | 194 |  |
| Thai-Myanmar | TM54 | C580Y | 288 | 194 | Hapl. 1 |
|  | TM58 | C580Y | 288 | 194 |  |
|  | TM48 | C580Y | 278 | 198 | Hapl. 2 |
|  | TM50 | C580Y | 278 | 198 |  |
|  | TM49 | C580Y | 278 | 202 | Hapl. 3 |
|  | TM75 | C580Y | 263 | 198 | Hapl. 4 |
|  | TM81 | C580Y | 263 | 198 |  |
|  | TM67 | C580Y | 263 | 194 | Hapl. 5 |
|  | TM72 | C580Y | 263 | 194 |  |
|  | TM78 | C580Y | 263 | 194 |  |
| China-Myanmar | TEC43 | C580Y | 263 | 194 | Hapl. 5 |
|  | CM09P-15 | C580Y | 278 | 198 | Hapl. 2 |
|  | CM09-50 | wild | 288 | 202 | Hapl. 6 |
|  | CM09P-6 | wild | 284 | 202 | Hapl. 7 |
|  | CM09P-18 | wild | 280 | 198 | Hapl. 8 |
|  | CM09M-13 | wild | 280 | 194 | Hapl. 9 |
|  | CM09-42 | wild | 280 | 194 |  |
|  | CM08-70 | wild | 278 | 194 | Hapl. 10 |
|  | CM09N-72 | wild | 276 | 194 | Hapl. 11 |
|  | CM09-20 | wild | 276 | 194 |  |
|  | CM08-20 | wild | 274 | 194 | Hapl. 12 |
|  | CM09-59 | wild | 263 | 202 | Hapl. 13 |

**Supplementary Table S4. SNPs in *arps10*, *fd* and *pph* among the isolates with R539T from China-Myanmar border, Thailand-Myanmar border and Thailand-Cambodia border.**

| sampling sites | sample | *arps10*  (G631A) | *fd*  (C1001A) | *pph*  (G3469C) |
| --- | --- | --- | --- | --- |
| China-Myanmar | CM08-27 | **G** | **C** | **G** |
|  | CM08-28 | **G** | **C** | **G** |
|  | CM08-35 | G | C | G |
|  | CM10-3 | **G** | **C** | **G** |
|  | CM10-31 | **G** | **C** | **G** |
|  | CM10-45 | **G** | **C** | **G** |
|  | CM10-13 | **G** | **C** | **G** |
|  | CM10-22 | **G** | **C** | **G** |
|  | CM09-43 | **G** | **C** | **G** |
|  | CM13-46 | **G** | **C** | **G** |
|  | CM10-27 | **G** | **A** | **G** |
|  | CM10-30 | **G** | **A** | **G** |
|  | CM10-35 | **G** | **A** | **C** |
|  | CM10-6 | **G** | **C** | **C** |
|  | CM10-21 | **G** | **C** | **C** |
|  | CM09-53 | **G** | **C** | **C** |
| Thai-Cambodia | TC5 | **A** | **A** | **C** |
|  | TC15 | **A** | **A** | **C** |
|  | TC34 | **A** | **A** | **C** |
|  | TC39 | **A** | **A** | **C** |
|  | TC40 | **A** | **A** | **C** |
|  | TC44 | **A** | **A** | **C** |
| Thai-Myanmar | TM46 | **A** | **A** | **C** |
|  | TM89 | **A** | **A** | **C** |

**Supplementary Table S5. Information of the isolates with parasite clearance time data.**

| **Sample ID** | **Sampling Sites** | **K13 mutation** | **Parasites Clearance Time** | | |
| --- | --- | --- | --- | --- | --- |
|  |  |  | **24h** | **48h** | **72h** |
| TC-31 | Thai-Cambodia | C580Y | ＋ | ＋ | ＋ |
| TC-32 | Thai-Cambodia | C580Y | ＋ | ＋ | ＋ |
| TC-33 | Thai-Cambodia | C580Y | ＋ | － | － |
| TC-34 | Thai-Cambodia | R539T | ＋ | ＋ | ＋ |
| TC-35 | Thai-Cambodia | C580Y | ＋ | ＋ | ＋ |
| TC-36 | Thai-Cambodia | C580Y | ＋ | ＋ | ＋ |
| TC-37 | Thai-Cambodia | C580Y | ＋ | ＋ | ＋ |
| TC-38 | Thai-Cambodia | G538V | ＋ | ＋ | ＋ |
| TC-39 | Thai-Cambodia | R539T | ＋ | ＋ | ＋ |
| TC-40 | Thai-Cambodia | R539T | ＋ | ＋ | ＋ |
| TC-41 | Thai-Cambodia | C580Y | ＋ | ＋ | ＋ |
| TC-42 | Thai-Cambodia | C580Y | ＋ | ＋ | ＋ |
| TC-43 | Thai-Cambodia | C580Y | ＋ | ＋ | ＋ |
| TC-44 | Thai-Cambodia | R539T | ＋ | ＋ | ＋ |
| TC-45 | Thai-Cambodia | C580Y | ＋ | ＋ | ＋ |
| TC-46 | Thai-Cambodia | R539T | ＋ | ＋ | ＋ |
| TC-47 | Thai-Cambodia | W | ＋ | ＋ | ＋ |
| CM08-1 | China-Myanmar | W | ＋ | － | － |
| CM08-2 | China-Myanmar | W | ＋ | － | － |
| CM08-3 | China-Myanmar | W | ＋ | － | － |
| CM08-4 | China-Myanmar | W | ＋ | － | － |
| CM08-5 | China-Myanmar | W | ＋ | － | － |
| CM08-7 | China-Myanmar | F446I | ＋ | － | － |
| CM08-8 | China-Myanmar | W | ＋ | － | － |
| CM08-9 | China-Myanmar | W | ＋ | － | － |
| CM08-10 | China-Myanmar | F446I | ＋ | － | － |
| CM08-11 | China-Myanmar | F446I | ＋ | － | － |
| CM08-12 | China-Myanmar | W | ＋ | － | － |
| CM08-13 | China-Myanmar | W | ＋ | － | － |
| CM08-15 | China-Myanmar | W | ＋ | － | － |
| CM08-16 | China-Myanmar | W | ＋ | － | － |
| CM08-17 | China-Myanmar | W | ＋ | － | － |
| CM08-18 | China-Myanmar | W | ＋ | － | － |
| CM08-19 | China-Myanmar | W | ＋ | － | － |
| CM08-20 | China-Myanmar | W | ＋ | － | － |
| CM08-21 | China-Myanmar | W | ＋ | － | － |
| CM08-22 | China-Myanmar | W | ＋ | － | － |
| CM08-24 | China-Myanmar | W | ＋ | － | － |
| CM08-25 | China-Myanmar | F446I | ＋ | － | － |
| CM08-26 | China-Myanmar | F446I | ＋ | － | － |
| CM08-27 | China-Myanmar | R539T | ＋ | ＋ | ＋ |
| CM08-28 | China-Myanmar | R539T | ＋ | ＋ | － |
| CM08-29 | China-Myanmar | W | ＋ | ＋ | － |
| CM08-30 | China-Myanmar | F446I | ＋ | － | － |
| CM08-32 | China-Myanmar | F446I | ＋ | － | － |
| CM08-33 | China-Myanmar | W | ＋ | － | － |
| CM08-34 | China-Myanmar | P574L | ＋ | － | － |
| CM08-35 | China-Myanmar | R539T | ＋ | － | － |
| CM08-36 | China-Myanmar | F446I | ＋ | ＋ | － |
| CM08-37 | China-Myanmar | F446I | ＋ | － | － |
| CM08-38 | China-Myanmar | F446I | ＋ | － | － |
| CM08-39 | China-Myanmar | P443S | ＋ | ＋ | － |
| CM08-40 | China-Myanmar | W | ＋ | － | － |
| CM08-41 | China-Myanmar | W | ＋ | － | － |
| CM08-42 | China-Myanmar | A676D | ＋ | － | － |
| CM08-43 | China-Myanmar | F446I | ＋ | ＋ | － |
| CM08-45 | China-Myanmar | F446I | ＋ | － | － |
| CM08-46 | China-Myanmar | P574L | ＋ | － | － |
| CM08-47 | China-Myanmar | W | ＋ | ＋ | － |
| CM08-48 | China-Myanmar | F446I | ＋ | － | － |
| CM08-49 | China-Myanmar | W | ＋ | － | － |
| CM08-51 | China-Myanmar | W | ＋ | － | － |
| CM08-52 | China-Myanmar | A481T | ＋ | － | － |
| CM08-53 | China-Myanmar | W | ＋ | － | － |
| CM08-54 | China-Myanmar | F446I | ＋ | － | － |
| CM08-55 | China-Myanmar | W | ＋ | － | － |
| CM08-57 | China-Myanmar | W | ＋ | － | － |
| CM08-58 | China-Myanmar | W | ＋ | － | － |
| CM08-60 | China-Myanmar | A676D | ＋ | ＋ | － |
| CM08-61 | China-Myanmar | W | ＋ | － | － |
| CM08-62 | China-Myanmar | W | ＋ | － | － |
| CM08-64 | China-Myanmar | F446I | ＋ | － | － |
| CM08-66 | China-Myanmar | G533A | ＋ | － | － |
| CM08-67 | China-Myanmar | F446I | ＋ | ＋ | ＋ |
| CM08-68 | China-Myanmar | W | ＋ | ＋ | － |
| CM08-69 | China-Myanmar | W | ＋ | － | － |
| CM08-70 | China-Myanmar | W | ＋ | － | － |
| CM08-71 | China-Myanmar | F446I | ＋ | － | － |
| CM08-72 | China-Myanmar | W | ＋ | － | － |
| CM08-73 | China-Myanmar | F446I | ＋ | － | － |
| CM08-74 | China-Myanmar | F446I | ＋ | － | － |
| CM09-2 | China-Myanmar | F446I | ＋ | ＋ | ＋ |
| CM09-3 | China-Myanmar | W | ＋ | － | － |
| CM09-4 | China-Myanmar | F446I | ＋ | － | － |
| CM09-6 | China-Myanmar | F446I | － | － | － |
| CM09-9 | China-Myanmar | W | ＋ | － | － |
| CM09-10 | China-Myanmar | W | － | － | － |
| CM09-12 | China-Myanmar | W | ＋ | － | － |
| CM09-17 | China-Myanmar | W | － | － | － |
| CM09-18 | China-Myanmar | W | ＋ | － | － |
| CM09-21 | China-Myanmar | F446I | ＋ | ＋ | － |
| CM09-23 | China-Myanmar | F446I | ＋ | ＋ | ＋ |
| CM09-27 | China-Myanmar | W | ＋ | － | － |
| CM09-32 | China-Myanmar | F446I | ＋ | ＋ | ＋ |
| CM09-33 | China-Myanmar | W | ＋ | ＋ | ＋ |
| CM09-36 | China-Myanmar | F446I | ＋ | － | － |
| CM09-37 | China-Myanmar | F446I | ＋ | － | － |
| CM09-38 | China-Myanmar | W | ＋ | － | － |
| CM09-40 | China-Myanmar | P574L | ＋ | ＋ | － |
| CM09-42 | China-Myanmar | W | ＋ | ＋ | － |
| CM09-45 | China-Myanmar | F446I | ＋ | ＋ | ＋ |
| CM09-46 | China-Myanmar | F446I | ＋ | ＋ | ＋ |
| CM09-47 | China-Myanmar | W | ＋ | － | － |
| CM09-48 | China-Myanmar | W | ＋ | ＋ | ＋ |
| CM09-50 | China-Myanmar | W | ＋ | ＋ | － |
| CM09-51 | China-Myanmar | F446I | ＋ | ＋ | － |
| CM09-52 | China-Myanmar | W | ＋ | － | － |
| CM09-54 | China-Myanmar | F446I | ＋ | － | － |
| CM09-55 | China-Myanmar | A676D | ＋ | － | － |
| CM09-56 | China-Myanmar | W | － | － | － |
| CM09-58 | China-Myanmar | F446I | ＋ | － | － |
| CM09-59 | China-Myanmar | W | ＋ | － | － |

W: wild type, ND: not detected

＋: positive, －: negative

**Supplementary Figure S1. Frequency of the *Kelch13* mutant alleles along the China-Myanmar border from 2007 to 2013**. All mutant alleles that carry a single non-synonymous SNP are shown in color, while those in yellow indicate the wild type allele.


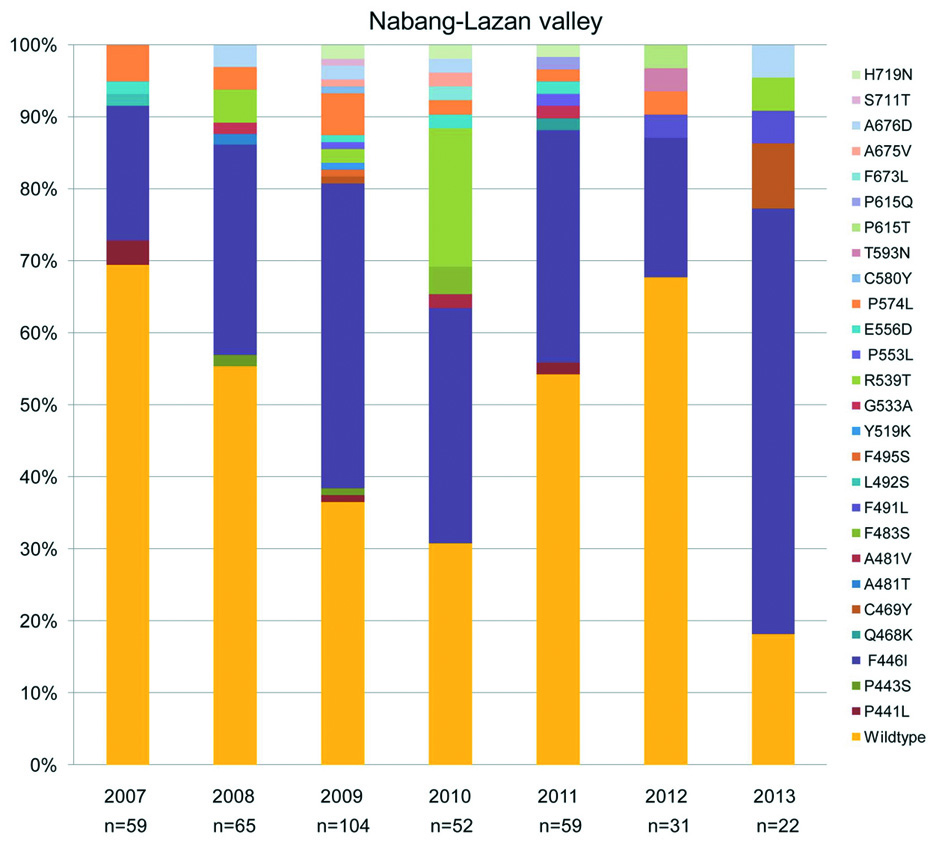


**Supplementary Method**

**Collection of blood samples**

The isolates from ‘Nabang-Lazan valley’ were obtained mostly from venous blood samples. Samples from other areas were obtained from finger-prick blood and absorbed onto Whatman filter paper. The filter papers were thoroughly dried and stored in a sealed plastic bag. The venous blood samples collected into heparin sodium vacutainers (medical instrument factory of Liuyang city of Hunan province, China) were cryopreserved in liquid nitrogen and delivered to the laboratory in Shanghai until use.

**Ring stage survival assay (RSA0-3h)**

The 0-3 hour post-invasion, ring stage parasites were exposed to 700 nM dihydroartemisinin (Sigma-Aldrich, USA) for six hours. They were then washed with normal medium to remove the drug before being cultured with the complete medium at 37°C for additional 66 hours. The same protocol with the ART-exposure step replaced with six hours exposure to 0.049% ethanol was applied to a control group. Using a microscope we counted the proportion of viable parasites that developed into second-generation parasites with normal morphology. Survival rate was calculated as follows: *Ps/Pc* × 100%, where *Pc* is the parasitemia in the control group and *Ps* is the parasitemia in the treatment group.
